# *BRAF* Expression and Copy Number Alterations Predict Unfavorable Tumor Features and Adverse Outcomes in Patients With Breast Cancer

**DOI:** 10.1155/2024/6373900

**Published:** 2024-05-30

**Authors:** Yazan R. Alhamdan, Nehad M. Ayoub, Sara K. Jaradat, Aymen Shatnawi, Rami J. Yaghan

**Affiliations:** ^1^ Department of Clinical Pharmacy Faculty of Pharmacy Jordan University of Science and Technology, PO Box 3030, Irbid 22110, Jordan; ^2^ Department of Drug Discovery and Biomedical Sciences College of Pharmacy Medical University of South Carolina, 70 President St., Charleston, South Carolina 29425, USA; ^3^ Department of Surgery College of Medicine and Medical Sciences Arabian Gulf University, Road 2904, Building 293, Manama, Bahrain; ^4^ Department of General Surgery and Urology Faculty of Medicine Jordan University of Science and Technology, PO Box 3030, Irbid 22110, Jordan

## Abstract

**Background:** The role of BRAF in breast cancer pathogenesis is still unclear. To address this knowledge gap, this study is aimed at evaluating the impact of BRAF gene expression and copy number alterations (CNAs) on clinicopathologic characteristics and survival in patients with breast cancer.

**Methods:** The Molecular Taxonomy of Breast Cancer International Consortium (METABRIC) dataset was obtained from the cBioPortal public domain. Tumoral *BRAF* mRNA expression and CNAs along with demographic and tumor data for patients with breast cancer were retrieved. The association of *BRAF* expression and CNAs with breast cancer clinicopathologic characteristics was analyzed. The impact of *BRAF* mRNA expression on the overall survival of patients was assessed using Kaplan–Meier survival analysis.

**Results:** BRAF gene mRNA log intensity expression was positively correlated with tumor size and the Nottingham Prognostic Index (NPI) (*p* < 0.001). Alternatively, BRAF gene expression was negatively correlated with the age at diagnosis (*p* = 0.003). The average *BRAF* mRNA expression was significantly higher in premenopausal patients, patients with high tumor grade, hormone receptor–negative status, and non-luminal tumors compared to postmenopausal patients, patients with low-grade, hormone receptor–positive, and luminal disease. *BRAF* gain and high-level amplification copy numbers were significantly associated with higher NPI scores and larger tumor sizes compared to neutral copy number status. Survival analysis revealed no discernible differences in overall survival for patients with low and high *BRAF* mRNA expression.

**Conclusion:** High *BRAF* mRNA expression as well as the gain and high-level amplification copy numbers were associated with advanced tumor characteristics and unfavorable prognostic factors in breast cancer. BRAF could be an appealing target for the treatment of premenopausal patients with hormone receptor–negative breast cancer.

## 1. Introduction

Breast cancer is the most common malignancy among women, accounting for about 30% of newly diagnosed cancer cases in the United States and 12% of global new annual cancer cases as of 2021 [1, 2]. Breast cancer is a heterogeneous disease at both the molecular and clinical levels. It comprises various biological entities and distinct histopathologic features known to have different morphological characteristics and clinical outcomes [3, 4]. According to comprehensive gene expression profiling, six major molecular subtypes of breast cancer have been revealed: normal breast-like, luminal A, luminal B, human epidermal growth factor receptor 2 (HER2)-enriched, claudin-low, and basal-like [5]. Luminal breast cancers are hormone receptor–positive and respond to hormonal therapies. HER2-enriched breast cancer is characterized by the amplification of the *HER2/ERBB2* oncogene and responds to targeted anti-HER2 therapy [6]. Basal-like tumors are predominantly triple-negative and lack the expression of hormone receptors and HER2. As a result, chemotherapy remains the standard treatment in patients with basal-like disease [6]. In the last decade, it is undeniable that breast cancer management and patient outcomes have improved remarkably; however, the exact mechanisms driving the development and progression of breast cancer remain largely unknown. Besides, the emergence of therapeutic resistance to available targeted therapies is on the rise, adding another layer of complexity to effective treatment and achieving optimal outcomes in breast cancer [7]. Thus, exploring new molecular targets is a high priority in breast cancer research.

The mitogen-activated protein kinase (MAPK) cascade is a crucial signaling hub regulating several cellular functions including cell proliferation, differentiation, and apoptosis [8, 9]. The RAS/RAF/MAPK pathway is frequently dysregulated in various types of human cancer, and several mutations were detected within the intracellular elements of this pathway known for their tumorigenic potential [10, 11]. RAF proteins are a family of serine/threonine kinases [12]. Three distinct RAF isoforms were identified in mammalian cells: ARAF, BRAF, and CRAF [12]. Among the RAF kinase family, the BRAF protein is a principal regulator of the MAPK signaling pathway and is encoded by the *BRAF* protooncogene located on chromosome 7q34 [13]. Mutated *BRAF* mediates oncogenic activity that constitutively promotes cell proliferation [14]. *BRAF* mutations are frequently detected in melanoma and were identified in arrays of cancers including colorectal cancer, non–small cell lung carcinoma, non–Hodgkin lymphoma, hairy cell leukemia, and thyroid cancer [14–17]. *BRAF* V600E is a notable mutation of the BRAF gene and the most identified among all reported *BRAF* mutations [18]. The V600E mutation increases the activity of the BRAF kinase [19]. Several studies demonstrated the association between the *BRAF* V600E mutation with adverse clinicopathologic features and poor outcomes in tumors such as colorectal and thyroid cancers [20–22]. *BRAF* mutations were also described in breast cancer, with the V600E being the most reported [19]. Köhler et al. showed that BRAF expression and activation are important for tumor initiation and lung metastasis in an animal model of breast cancer [23]. In addition to point mutations, aberrant BRAF gene expression has been demonstrated in human cancers. A recent analysis of the TCGA dataset showed high BRAF mRNA expression in several cancer types including cervical, colon, esophageal, hepatocellular, lung, gastric, and uterine cancers as well as cholangiocarcinoma [24]. Although growing evidence has investigated the expression of BRAF and its role in several human cancers, the impact of *BRAF* expression in breast cancer is less clear. Therefore, this study is aimed at comprehensively analyzing *BRAF* expression and gene copy number status in breast cancer and its association with clinicopathologic characteristics and survival.

## 2. Methods

### 2.1. The Molecular Taxonomy of Breast Cancer International Consortium (METABRIC) Dataset

The METABRIC dataset was generated based on clinical and genomic data for patients with primary breast tumors from five different hospitals and/or research centers in the United Kingdom and Canada [25]. The dataset contains the molecular profiles of more than 2000 breast tumors along with long-term follow-up data to enable the analysis of the association between cancer genomics/transcriptomics and the clinical attributes and disease outcomes in patients with breast cancer [25]. The METABRIC dataset is freely available from the cBio cancer genomics portal (cBioPortal) which is an open-access resource for the interactive exploration of multidimensional cancer genomics datasets [26, 27]. Additionally, the METABRIC dataset contains pertinent patient demographic and clinical data such as the age at diagnosis, menopausal status, Nottingham Prognostic Index (NPI), and type of treatment received by patients including surgery, hormonal therapy, chemotherapy, and/or radiotherapy. The dataset also provides detailed information regarding tumor clinicopathologic characteristics such as size, grade, stage, histological subtype, the number of positive lymph nodes, receptor status, and the molecular subtype. Furthermore, the survival time and survival status of patients are available.

The METABRIC dataset includes microarray gene expression profile analysis and putative copy number alterations (CNAs) for hundreds of genes available in the dataset. The expression values for each gene in the cohort are presented as log2 intensity values [28]. For this study, BRAF mRNA expression intensities were obtained from the dataset and were available for 1904 patients. Besides, *BRAF* CNAs were available for 2173 patients in this cohort. The values of CNAs were −1: hemizygous deletion; 0: neutral (no change); 1: gain; and 2: high-level amplification.

The downloaded demographic, clinical, and tumor data from the METABRIC dataset were cleaned to avoid any inconsistencies or entry errors. Afterward, the variables were coded and uploaded to the statistical software. To perform survival analysis, the continuous gene expression data were converted to categorical data. The mRNA expression of *BRAF* was divided into *low* and *high* expression categories based on the mean expression value, in which patients with mRNA expression levels equal to or less than the mean value were categorized to have a *low* expression status, while those with expression levels greater than the mean value were indicated to have a *high* expression status. Furthermore, the dichotomization of some categorical variables was considered for association analysis and was performed in advance of conducting statistical analysis to avoid a small sample size. The categories of these tumor variables were selected using cut points previously reported [29]. Therefore, tumor grade was categorized as grade I/II and grade III. The TNM stage was dichotomized as early (stage I/II) and advanced (stage III/IV). The molecular subtype was grouped as luminal (luminal A and luminal B) and non-luminal (HER2-enriched, basal-like, claudin-low, and normal-like) tumors.

### 2.2. Statistical Analysis

The data were analyzed using the SPSS statistical package, version 28.0 (IBM Corp., Armonk, NY). Continuous variables are presented as the mean ± standard deviation for normally distributed variables and as the median and interquartile range (IQR) for non-normally distributed variables. The categorical variables are presented as frequencies and percentages. The independent sample *t*-test was used to compare the means of the two groups. For normally distributed variables, one-way analysis of variance (ANOVA) was used for comparisons between multiple independent groups, followed by the Benjamini–Hochberg (false discovery rate (FDR)) procedure to control the expected proportion of false discoveries. For nonnormal distribution variables, the nonparametric Kruskal–Wallis analysis for multiple independent groups was used. Pearson's correlation test was applied to assess the correlations between the continuous variables, and the chi-square test of independence was used to assess associations between categorical variables. The Kaplan–Meier survival curves were generated for patients according to the BRAF gene expression status using GraphPad Prism, version 8.0.1, software (GraphPad Software, San Diego, CA). Cox proportional hazard models were fitted with overall survival as the outcome. All *p* values were two-sided, and differences were considered statistically significant at *p* < 0.05.

## 3. Results

### 3.1. Description of Patients With Breast Cancer in the METABRIC Dataset

The demographic and clinicopathologic characteristics of the METABRIC dataset were previously described [29]. The mean age at diagnosis was 60.4 ± 13.0 years (range 21.9–96.3), and the average NPI score was 4.0 ± 1.2 (range 1.0–7.2). The median tumor size was 22.41 mm (IQR, 17.0–30.0). Most patients were postmenopausal (78.6%), and invasive ductal carcinoma was the most reported histopathologic type (76.2%). Half of the patients (50.2%) had high-grade carcinoma. Luminal A (35.5%) and luminal B (24.1%) were the most frequent molecular subtypes.

### 3.2. Expression of BRAF in Patients With Breast Cancer

The mean *BRAF* mRNA expression log intensity was 5.93 ± 0.3 (range 5.2–7.6) (Table 1). Among 1904 patients, 1087 (57.1%) had low *BRAF* expression and 817 (42.9%) had high expression. Most patients (76.5%) had no changes in the *BRAF* copy number. The gain was the most frequently reported *BRAF* CNA (11.1%), followed by hemizygous deletion (10.4%) and high-level amplification (2.0%) (Table 1). None of the patients in this cohort had a *BRAF* homozygous deletion.

### 3.3. BRAF mRNA Expression and Clinicopathologic Characteristics of Patients With Breast Cancer

BRAF gene expression correlated positively with the tumor size and the NPI scores (*r* = 0.092 and *r* = 0.112 (*p* < 0.001), respectively) (Table 2). Besides, an inverse correlation was shown between the age at diagnosis and *BRAF* mRNA level (*r* = −0.067; *p* = 0.003, Table 2). No correlation was found between the number of positive lymph nodes and the levels of *BRAF* mRNA transcript in patients.

**Figure 1:**
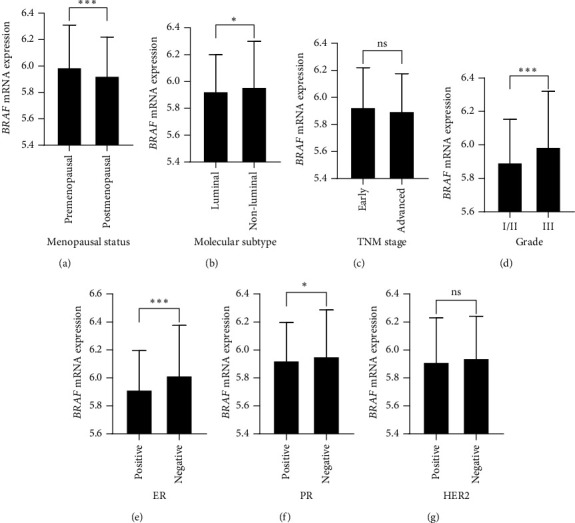
*BRAF* mRNA expression based on clinicopathologic characteristics of patients with breast cancer. The expression of *BRAF* according to (a) menopausal status, (b) molecular subtype, (c) TNM stage, (d) grade, (e) ER status, (f) PR status, and (g) HER2 status in patients with breast cancer. Bars represent mean mRNA gene expression log intensity ± standard deviation. ^∗^*p* < 0.05 and ^∗∗∗^*p* < 0.001. ER, estrogen receptor; HER2, human epidermal growth factor receptor 2; ns, no statistically significant difference according to independent sample t-test; PR, progesterone receptor.

The mean mRNA expression of *BRAF* was significantly higher in premenopausal compared to postmenopausal patients (*p* < 0.001, Figure 1(a)). *BRAF* expression was significantly elevated in patients diagnosed with non-luminal tumors compared to those harboring the luminal subtype (*p* = 0.021, Figure 1(b)). Patients with high-grade carcinoma had significantly higher expression of the BRAF gene compared to their counterparts with low-to-moderate grade tumors (*p* < 0.001, Figure 1(d)). Further, *BRAF* mRNA levels were significantly higher in patients harboring hormone receptor–negative tumors compared with those with hormone receptor–positive status (*p* < 0.001, Figures 1(e) and 1(f)). However, no significant differences in *BRAF* expression were found according to tumor stage or HER2 status (Figures 1(c) and 1(g)).

### 3.4. BRAF CNAs and Clinicopathologic Characteristics of Patients With Breast Cancer

**Figure 2:**
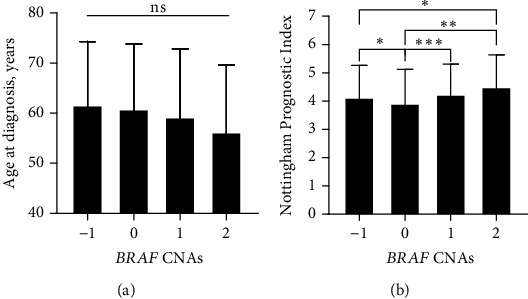
Age and Nottingham Prognostic Index (NPI) based on *BRAF* CNAs in patients with breast cancer. Bars represent the mean (a) age at diagnosis and (b) NPI scores in patients with breast cancer stratified based on the *BRAF* CNA group. Bars represent mean mRNA gene expression log intensity ± standard deviation. CNA values: −1, hemizygous deletion; 0, neutral (no change); 1, gain; and 2, high-level amplification. ^∗^*p* < 0.05, ^∗∗^*p* < 0.01, and ^∗∗∗^*p* < 0.001. The *p* values represent the Benjamini–Hochberg (FDR) adjusted *p* values. CNAs, copy number alterations; ns, no statistically significant difference according to one-way ANOVA.

**Figure 3:**
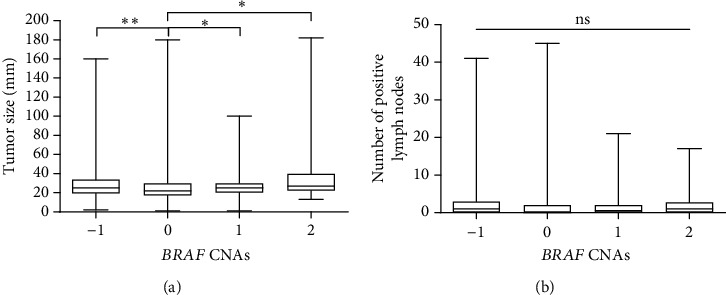
Tumor size and lymph nodes based on *BRAF* CNAs in patients with breast cancer. Boxplots represent the median (a) tumor size and (b) number of positive lymph nodes in patients with breast cancer at the time of diagnosis stratified based on the *BRAF* CNA group. The boxes represent the 25^th^ and 75^th^ percentiles, whereas the bars represent minimum and maximum values. CNA values: −1, hemizygous deletion; 0, neutral (no change); 1, gain; and 2, high-level amplification. ^∗^*p* < 0.05 and ^∗∗^*p* < 0.01. CNAs, copy number alterations; ns, no statistically significant difference according to the Kruskal–Wallis analysis of variance.

Normality analysis revealed that the age of patients and the NPI scores were normally distributed, while the tumor size and the number of positive lymph nodes were non-normally distributed. Accordingly, a one-way ANOVA was used to compare the age of patients and the NPI scores, while a nonparametric Kruskal–Wallis analysis was used to compare the tumor size and the number of lymph nodes among the different CNA groups. While no statistically significant differences in the age of patients were observed, there is a trend of reduced age at diagnosis for patients with gain and high-level amplification CNAscompared to patients who had neutral copy number or hemizygous deletion (*p* = 0.064 and *p* = 0.068, respectively, Figure 2(a)). Tumors with *BRAF* high-level amplification had higher NPI scores compared to those with neutral copy numbers and patients with hemizygous deletion (*p* = 0.006 and *p* = 0.04, respectively, Figure 2(b)). Besides, the mean NPI scores were higher in patients with gain CNA compared to those with no change (*p* < 0.001, Figure 2(b)). The distribution of the tumor size was significantly different among the CNA groups (*p* < 0.001, Figure 3(a)). Tumors with *BRAF* high-level amplification and gain had significantly higher median tumor sizes compared to those with neutral copy numbers (*p* = 0.019 and *p* = 0.013, respectively). No differences in the median number of positive lymph nodes were detected across the different *BRAF* CNAs in patients with breast tumors (*p* = 0.305, Figure 3(b)).

*BRAF* CNAs showed a significant association with tumor grade, hormone receptors, and molecular subtype (Table 3). A greater proportion of patients who have *BRAF* gain (13.8%) or high-level amplification (3.3%) are presented with high-grade tumors compared to those with low-to-moderate grade tumors (*p* < 0.001). Likewise, a higher proportion of patients with hormone receptor–negative status had gain and high-level amplification CNAs compared to patients with hormone receptor–positive disease (*p* < 0.001, Table 3). Gain and high-level amplification CNAs were also associated with an increased number of non-luminal tumors compared to patients with luminal cancers (*p* < 0.001, Table 3).

### 3.5. BRAF Expression and Overall Survival of Patients With Breast Cancer

**Figure 4:**
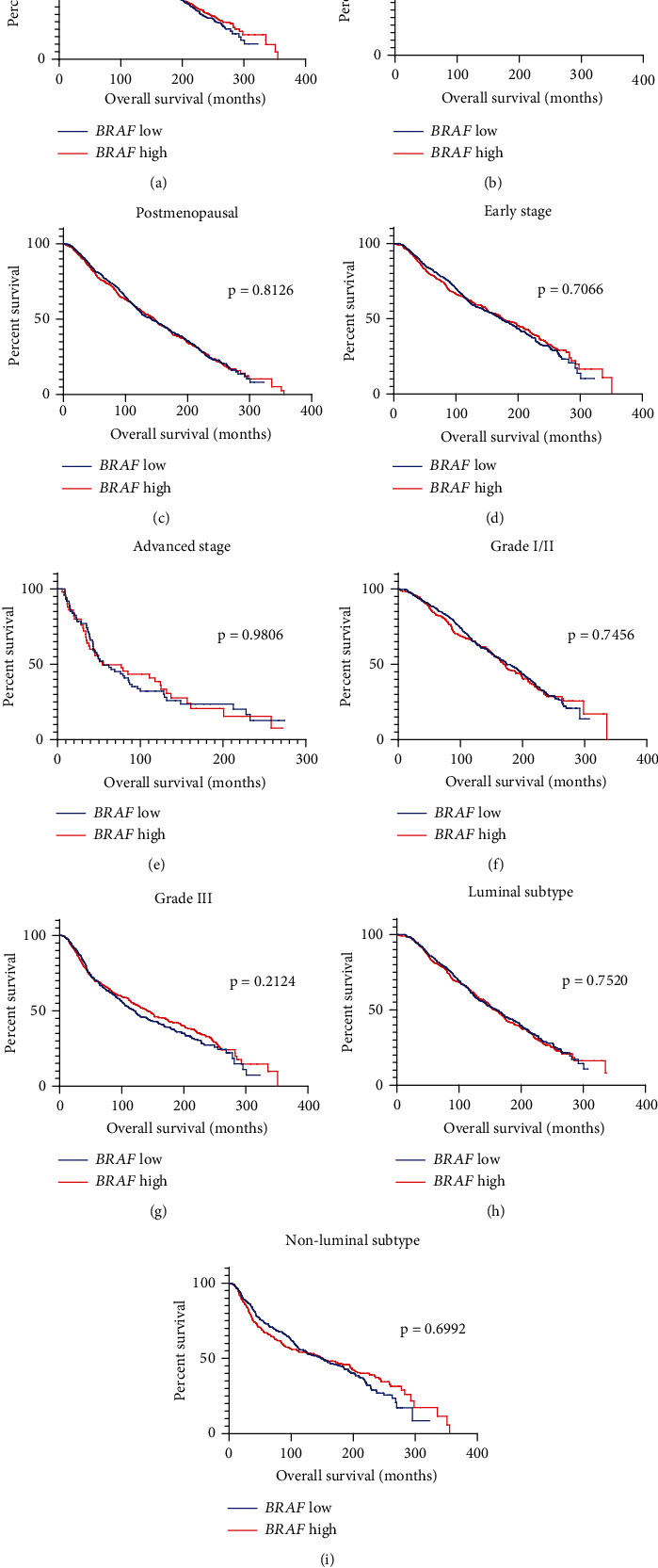
Overall survival according to *BRAF* expression status in patients with breast cancer. Kaplan–Meier survival analysis for high and low *BRAF* expression in (a) all patients, (b) premenopausal, (c) postmenopausal, (d) early-stage, (e) advanced-stage, (f) grade I/II, (g) grade III, (h) luminal, and (i) non-luminal disease.

Kaplan–Meier survival analysis revealed no significant differences in overall survival between low- and high-expressing groups of *BRAF* among patients with breast cancer (median survival 155.37 vs. 153.87 months, *p* = 0.888, respectively, Figure 4(a)). Among premenopausal patients, there was a trend towards greater survival in patients with *BRAF* high expression compared to those with low expression, though it did not reach statistical significance (median survival 282.57 vs. 228.1 months, respectively, *p* = 0.888, Figure 4(b)). In postmenopausal cases, the median survival times between the *BRAF* low- and high-expressing groups were very close and the survival curves were not significantly different (median survival 142.67 vs. 147.37 months, respectively, *p* = 0.8126, Figure 4(c)). Upon further stratification of data based on clinicopathologic and prognostic characteristics, survival curves for patients with low and high expression of *BRAF* were not significantly different according to tumor stage, grade, and molecular subtype (Figures 4(d), 4(e), 4(f), 4(g), 4(h), and 4(i)).

## 4. Discussion

Breast cancer is a highly heterogeneous disease consisting of clinically and pathologically distinct subtypes [30]. The presence of multiple molecular subtypes within a primary tumor complicates treatment decisions and clinical outcomes. The use of gene expression signature–based tools has advanced our understanding of genes and intracellular signaling pathways that could impact tumor development and progression [30]. In this regard, analyzing large-scale data could deliver a more comprehensive understanding of the various properties of genes, proteins, or biomolecules [31]. *BRAF* is a protooncogene known for its tumorigenic activity in melanoma, hairy cell leukemia, non–Hodgkin lymphoma, thyroid, ovarian, lung, and colorectal cancers [32]. Nevertheless, the impact of *BRAF* expression on the development and progression of breast cancer is inconclusive. Our analysis of the METABRIC dataset revealed that *BRAF* expression and CNAs were associated with adverse clinicopathologic tumor features and poor prognosticators in patients with breast cancer.

While age is positively associated with the increased risk of breast cancer, it is estimated that 5%–7% of breast cancer cases are diagnosed in patients younger than 40 years of age [33]. Typically, breast cancer in younger patients is more aggressive, with higher rates of mortality and locoregional recurrence compared to older cases [33, 34]. Besides, younger patients are more likely to be present with unfavorable tumor features such as larger tumors, higher grade, more positive lymph nodes, hormone receptor negativity, HER2-overexpression, and a greater extent of lymphovascular invasion than older women [33, 34]. Also, familial factors and family history are frequently observed in patients under 40 years of age [34]. Findings from our analysis revealed an inverse correlation between *BRAF* mRNA expression and the age of patients at diagnosis. Further, the expression of *BRAF* was higher in premenopausal patients compared to postmenopausal cases. In a recent analysis by Alghanim et al., the expression of genes could be altered according to breast cancer menopausal status [35]. Accordingly, insights into the temporal links between genetic and epigenetic changes based on menopausal transitions could highly impact potential biomarkers related to breast cancer development [35]. Our study indicated a higher expression of *BRAF* in the younger premenopausal patients which could explain, at least in part, the genetic background for breast cancer in this patient population along with their advanced clinicopathologic features at diagnosis.

The NPI is a widely accepted prognostic tool that combines tumor size, nodal status, and histologic grade for patients with invasive carcinoma of the breast [36]. Patients with low NPI (2.02–2.4) have excellent 10-year survival rates [36]. *BRAF* mRNA expression correlated positively with NPI in this analysis supporting its adverse impact on the prognosis of patients with breast cancer. Additionally, *BRAF* expression was higher in patients with larger tumor sizes and higher grades. These findings are consistent with earlier studies in which BRAF was associated with larger tumor size in thyroid and skin cancers [37, 38]. Our findings revealed no association between *BRAF* mRNA levels with the stage of carcinoma and lymph node metastasis. Alternatively, BRAF expression was shown to be associated with lymph node metastasis in patients with melanoma and papillary thyroid carcinoma [39, 40]. Also, *BRAF* expression correlated with the pathological stage in colon adenocarcinoma, kidney renal clear cell carcinoma, lung squamous cell carcinoma, and ovarian serous cystadenocarcinoma [24]. Patients with breast cancer may have different characteristics that make *BRAF* expression less predictive of pathological stage and lymph node metastasis.

Traditional classification systems for the molecular profiling of breast cancer are largely determined based on the expression status of hormone receptors (estrogen receptors and progesterone receptors) and HER2 status [41]. Approximately 75% of breast cancers are luminal tumors characterized by hormone receptor positivity and a favorable prognosis. HER2-positive cancers are characterized by the overexpression of HER2 and are associated with a more aggressive tumor phenotype compared to luminal disease. Triple-negative tumors lack the expression of all three receptors and are known to have poor clinical outcomes [41]. Although this classification system is globally utilized for profiling and personalizing treatment plans in breast cancer, the classical histological analysis of tumors might not adequately address the complex genetic alterations mediating cancer development and progression [41]. Our findings revealed a remarkable association between *BRAF* expression, hormone receptor negativity, and the non-luminal tumor subtype. Clinically, few reports showed a favorable response of metastatic triple-negative breast cancer to the BRAF inhibitor, vemurafenib [42, 43]. Nevertheless, HER2 status was not associated with *BRAF* mRNA expression in our study. While the activation of the PI3K/Akt pathway is frequently linked to HER2-positive breast cancer, less effect is observed for BRAF expression on the tumor biology of this molecular subtype. However, the strong association of *BRAF* expression with the hormone receptor–negative and non-luminal subtypes can be employed as a marker of more aggressive tumor biology.

CNAs involve deletions or amplifications of fragments of genomic material that highly contribute to cancer development, progression, and therapeutic resistance [44]. *BRAF* CNAs were found in 23.5% of patients in this cohort, and gain was the most frequent alteration. *BRAF* copy gains have been associated with higher *BRAF* gene expression and protein levels in previous reports [45]. In this study, *BRAF* copy number gain and high-level amplification were associated with unfavorable clinicopathologic characteristics, including higher NPI scores, larger tumor size, high-grade tumors, hormone receptor–negative status, and non-luminal subtypes, compared to patients with no CNAs. In line with this, Ciampi, Zhu, and Nikiforov reported that *BRAF* copy number gain was associated with more invasive follicular thyroid carcinomas compared to tumors with no copy number change [46]. Further, *BRAF* amplification mediated resistance to MEK and BRAF inhibitors in colorectal cancer cells [47]. Interestingly, the cooccurrence of *BRAF* mutations and copy number gains was reported in human cancers. Sasaki et al. showed that increased *BRAF* copy number was correlated with *BRAF* V600E mutations in lung cancer patients [48]. Therefore, *BRAF* gain and high-level amplification CNAs can be employed as markers of unfavorable characteristics of breast cancer.

The expression status of the BRAF gene did not influence the overall survival of patients with breast cancer in this analysis of the METABRIC dataset. Stratification of patients according to menopausal status, tumor stage, grade, and molecular subtype revealed no significant change in survival among patients with low and high *BRAF* expression groups. In a recent study by Yi et al., higher BRAF expression was associated with a better prognosis and improved relapse-free survival in patients with invasive breast cancer [24]. Alternatively, Liu and Zhou indicated that higher BRAF expression is associated with better survival in hormone receptor–positive breast cancers but with worse survival and increased recurrence in hormone receptor–negative disease [49]. Increased BRAF expression was also associated with poor relapse-free survival in hepatocellular carcinoma, lung squamous cell carcinoma, and uterine corpus endometrial carcinoma [24]. An explanation for these findings may, in part, be associated with other factors influencing the overall survival, such as the type of cancer, the age of patients, the stage of disease, or genetic mutations. In addition, it is possible that the cutoffs used to define high versus low expression of BRAF were different from ours and impacted the survival results in our study. Besides, patients with worse prognostic factors will likely receive more aggressive treatment, masking the effect of *BRAF* expression status. In this context, Alghanim et al. proposed a pipeline to integrate multiomics data regarding gene expression, CNAs, and DNA methylation in breast cancer according to menopausal status [35]. Their data showed that RUNX1, PTEN, MAP3K1, and CDH1 had the highest impact in distinguishing survival curves of premenopausal and postmenopausal breast cancer [35]. In relation to this, ElKarami et al. showed that the UMAP embedding technique can best integrate the multiomics maps into the tumor prediction model [31].

## 5. Conclusion

Understanding the genes that might contribute to the advanced presentation and poor prognosis of breast cancer is particularly important to the development of novel therapeutic targets. Analysis of the METABRIC dataset revealed a significant role of the BRAF gene and its CNAs in relation to unfavorable clinicopathologic characteristics and poor prognosis in breast cancer. BRAF inhibitors could be a promising therapeutic option for a selected group of patients with breast cancer, calling for further exploration of the mutational profile of *BRAF* in breast cancer.

## Figures and Tables

**Table 1:** mRNA and copy number alterations of BRAF gene in patients with breast cancer.

**Characteristic**	**Value**
*BRAF* mRNA expression, log intensity	
Mean ± standard deviation	5.93 ± 0.3
Range	5.2–7.6
*BRAF* mRNA expression status^†^	*n* (%)
Low	1087 (57.1)
High	817 (42.9)
*BRAF* CNAs	*n* (%)
Homozygous deletion	0 (0.0)
Hemizygous deletion	225 (10.4)
Neutral/no change	1662 (76.5)
Gain	242 (11.1)
High-level amplification	44 (2.0)

*Note:* mRNA expression data were available for 1904 patients.

Abbreviation: CNAs, copy number alterations.

^†^Low expression: mRNA log intensity of ≤ 5.93; high expression: mRNA log intensity of > 5.93.

**Table 2:** Correlation of *BRAF* mRNA expression with clinicopathologic characteristics of patients with breast cancer.

**Characteristic**	***BRAF* mRNA expression, log intensity**	
	**r**	**p** **value**
Age at diagnosis (years)	−0.067	0.003∗
Nottingham Prognostic Index	0.112	< 0.001∗
Tumor size (mm)	0.092	< 0.001∗
Number of positive lymph nodes	0.033	0.148

Abbreviation: *r*, Pearson's correlation coefficient.

^*^indicates statistical significance.

**Table 3:** Association of *BRAF* copy number alterations with clinicopathologic characteristics in patients with breast cancer.

**Characteristic**	***BRAF* CNAs**				
	**Hemizygous deletion (** **n** = 225**)**	**Neutral (** **n** = 1662**)**	**Gain (** **n** = 242**)**	**High-level amplification (** **n** = 44**)**	**p** **value**
TNM stage					0.517
Early	148 (10.6)	1071 (76.5)	154 (11.0)	27 (1.9)	
Advanced	15 (11.0)	107 (78.7)	10 (7.4)	4 (2.9)	

Grade					< 0.001∗
I/II	87 (8.5)	836 (81.6)	94 (9.2)	8 (0.8)	
III	124 (11.8)	744 (71.1)	144 (13.8)	35 (3.3)	

ER					< 0.001∗
Positive	161 (10.0)	1275 (79.2)	153 (9.5)	20 (1.2)	
Negative	58 (11.9)	320 (65.6)	87 (17.8)	23 (4.7)	

PR					< 0.001∗
Positive	102 (9.8)	833 (80.1)	93 (8.9)	12 (1.2)	
Negative	100 (10.6)	687 (73.1)	127 (13.5)	26 (2.8)	

HER2					0.153
Positive	28 (11.3)	181 (73.3)	29 (11.7)	9 (3.6)	
Negative	174 (10.0)	1339 (77.3)	191 (11.0)	29 (1.7)	

Molecular subtype					0.005∗
Luminal	117 (10.0)	929 (79.1)	113 (9.6)	16 (1.4)	
Non-luminal	83 (10.4)	587 (73.5)	107 (13.4)	22 (2.8)	

*Note*: Data are presented as n (%). Pearson's chi-square test of independence. Luminal tumors include both luminal A and B tumors. Non-luminal tumors include HER2-enriched, basal-like, claudin-low, and normal-like tumors.

Abbreviations: CNAs, copy number alterations; ER, estrogen receptor; HER2, human epidermal growth factor receptor 2; PR, progesterone receptor.

^*^indicates statistical significance.

## Data Availability

The METABRIC dataset used for this analysis is freely accessible in the cBioPortal public domain (available at https://www.cbioportal.org/).
